# The World as an External Memory: The Price of Saccades in a Sensorimotor Task

**DOI:** 10.3389/fnbeh.2018.00253

**Published:** 2018-11-20

**Authors:** Andrew Melnik, Felix Schüler, Constantin A. Rothkopf, Peter König

**Affiliations:** ^1^Institute of Cognitive Science, University of Osnabrück, Osnabrück, Germany; ^2^Cognitive Interaction Technology – Excellence Center (CITEC), Bielefeld University, Bielefeld, Germany; ^3^Center for Cognitive Science, Institute of Psychology, Technische Universität Darmstadt, Darmstadt, Germany; ^4^Department of Neurophysiology and Pathophysiology, University Medical Center Hamburg-Eppendorf, Hamburg, Germany

**Keywords:** embodied cognition, working memory, short-term memory, sensorimotor task, adaptive behavior, visual sampling, saccadic eye movements, eye-tracking

## Abstract

Theories of embodied cognition postulate that the world can serve as an external memory. This implies that instead of storing visual information in working memory the information may be equally retrieved by appropriate eye movements. Given this assumption, the question arises, how we balance the effort of memorization with the effort of visual sampling our environment. We analyzed eye-tracking data in a sensorimotor task where participants had to produce a copy of a LEGO^®^-blocks-model displayed on a computer screen. In the unconstrained condition, the model appeared immediately after eye-fixation on the model. In the constrained condition, we introduced a 0.7 s delay before uncovering the model. The model disappeared as soon as participants made a saccade outside of the Model Area. To successfully copy a model of 8 blocks participants made saccades to the Model Area on average 7.9 times in the unconstrained condition and 5.2 times in the constrained condition. However, the mean duration of a trial was 2.9 s (14%) longer in the constrained condition even when taking into account the delayed visibility of the model. In this study, we found evidence for an adaptive shift in subjects’ behavior toward memorization by introducing a price for a certain type of saccades. However, the response is not adaptive; it is maladaptive, as memorization leads to longer overall performance time.

## Introduction

Theories of embodied cognition postulate that the world can serve as an external memory (Clark and Chalmers, 1998). “The feeling of the presence and richness of the visual world is a kind of illusion, created by availability of the information in this external store” (O’Regan, 1992). Under this view, instead of storing visual information in working memory the information may be equally retrieved by appropriate eye movements (Van Gompel, 2007).

Humans use eye fixations to serialize a process of solving a larger cognitive goal (Findlay and Gilchrist, 2003; Land and Tatler, 2009). The complexity of visual computations can be substantially reduced by decomposing a given task into simpler object identification and localization tasks, thereby making it computationally more tractable (Ballard et al., 1997). Saccadic eye movements allow incremental access to the immediately task-relevant information and, therefore, reflect cognitive events (Land and Hayhoe, 2001). Limited information retained from prior saccades is determined by what is currently relevant for the sensorimotor task (Ballard et al., 1997). A shared representational domain of certain products of perception and certain antecedents of action in the human brain (Melnik et al., 2017) can be the reason for this limitation. Effectively, humans trade the effort of memorization with the effort of active sampling of visual information (Inamdar and Pomplun, 2003; Droll and Hayhoe, 2007; Hardiess et al., 2008).

More complex sensorimotor acts (e.g., uttering a sentence, social behavior) are based on a sequence of sensory-motor primitives (e.g., eye movements) (Newell, 1994). Sequential actions require a buffer to store and operate over different features of sensory information (Ballard et al., 1992). Short-term memory temporarily holds a limited amount of information in an accessible state, and working memory is used to plan and carry out behavior (Cowan, 2008). Short-term memory has a capacity of 3–7 units and temporal decay limitations (Miller, 1956; Cowan, 2001, 2008; Vogel et al., 2001). This capacity limitation of short-term memory may not be a shortage, but a consequence of the cognitive architecture of the human brain that allows efficient searching, grouping, and processing of information. “The conception of working memory as the set of currently active pointers leads to a very simple interpretation of the tradeoffs between working memory load and eye movements, in which fixation can be seen as a choice of an external rather than an internal pointer” (Ballard et al., 1997). This reasoning predicts that the balance of memorization and active sampling might be shifted by making one or the other more effortful. In the present study, we test this prediction in a block copying task.

## Materials and Methods

### Participants

Twenty paid healthy volunteers (seven males), aged 19–27 years (mean = 22.7), participated in this study. All participants had normal (self-reported) or corrected-to-normal vision. We obtained written informed consent from all subjects before the experiment, and the protocol had been approved by the review board of the University of Osnabrück. Compensation for participation in the experiment was 7.5 Euros. The study was approved by the ethics committee of the University of Osnabrück.

### Stimuli and Task

**FIGURE 1:**
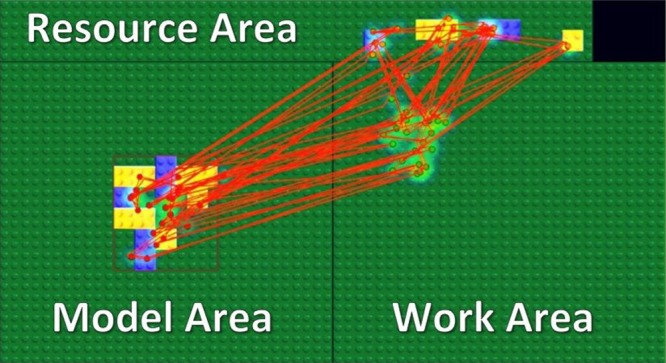
Experimental setup. We divided the screen area into three parts: Model Area, Work Area, and Resource Area. Participants had to copy a model of eight blocks as fast as possible. Red lines and dots represent saccades and fixations of a subject respectively in a single trial.

Our stimuli and task were motivated by a related study with a block-copying task (Ballard et al., 1995) and implemented in a gaze-contingent paradigm with eye and mouse tracking. In the experiment, we divided a computer screen into three main areas: the Model Area, the Work Area, and the Resource Area (Figure 1). A model always appeared in the center of the Model Area, and its location was outlined by the red rectangle shown in Figure 1. A model appeared only during fixations inside the Model Area and disappeared while fixations outside of the Model Area. Therefore, participants could not obtain visual information from peripheral vision while working in the Work Area or the Resource Area.

We asked participants to copy a model of eight blocks displayed in the Model Area by dragging and dropping blocks from the Resource Area into the Work Area. Participants were able to move a block by drag and drop and rotate it by a right click on a computer mouse. By clicking on a block of a specific size and color in the Resource Area, a new instance of that block was created and attached to a computer mouse cursor. Participants could dispose of a block picked up by mistake in the trash-area (black rectangle in the Resource Area). Each block was of one of two colors (blue or yellow) and one of two types (square or rectangle) (Figure 1).

### Unconstrained and Constrained Conditions

**FIGURE 2:**
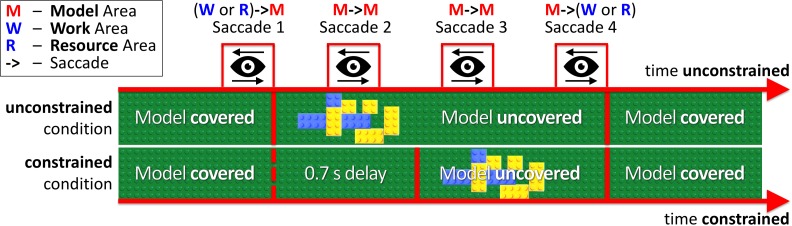
Relative timelines of unconstrained and constrained conditions (absolute time unconstrained ≠ time constrained). The example demonstrates Model visibility in the unconstrained and constrained conditions during four saccades (WM, MM, MM, MW). In the unconstrained condition, the model appeared immediately after eye-fixation in the Model Area (Saccade 1). In the constrained condition, we introduced a 0.7 s delay before uncovering the model. The MM bars in Figure 3 and MM column in Table 1 represent Saccades 2 and 3 in the current figure.

In our experimental paradigm, we introduced an additional price of saccades (penalty for visually sampling the block-model) as a 0.7 s delay for uncovering a block-model in the constrained condition (Figure 2 constrained condition). Thus, participants had to maintain eye fixations in the Model Area during the delay period for the model to appear. In the unconstrained condition (Figure 2 unconstrained condition), the model appeared immediately after eye-fixation in the Model Area. In both conditions, the block-model disappeared as soon as participants made a saccade outside of the Model Area.

The price of a certain type of saccades in the constrained condition was the central parameter in the design of our study. It is roughly triple the typical inter-saccadic interval. The price was designed to be high enough to motivate participants to avoid saccades into the Model Area, but without a high frustration for paying it. In a pilot experiment with a few independent participants, we explored different values for the delay-duration. In this study, we settled on the 0.7 s delay period, which appeared as a good compromise of achieving a high statistical power, large effect size, and maintaining natural viewing behavior. However, to better understand whether changes in viewing behavior follow a linear dependence or are more of a threshold effect, future studies should vary parameters of the model in smaller steps to elucidate the precise relation.

The design of the experiment and the provided instructions required from participants to be as fast as possible, yet to be accurate as well. Our goal was to allow participants to complete 90% of trials within a given duration of trials. In a pilot study, we recorded two participants without any limitations on the trial duration. Results from the pilot recordings suggested setting the maximum duration of trials in the unconstrained condition to 27 s. The duration of trials in the constrained condition was longer than in unconstrained condition to account for the delay periods (0.7 s). To determine a duration of trials in the constrained condition we computed the mean number of evoked delays (saccades into the Model Area) in the 90% of shortest trials, multiplied this number by the duration of the delay period (0.7 s) and added this to the duration of trials in the unconstrained condition. The mean number of penalties in the pilot recordings in the 90% fastest trials was 13.8 which resulted in a maximum duration of 37 s for constrained condition trials (27 s + 13.8 × 0.7 s = 36.66 s, or 37 s). Thus, we set the duration of trials to 27 s in the unconstrained condition and 37 s in the constrained condition. Trials ended automatically when participants correctly finished copying the entire model. Trials also ended when the model was not completely copied during the specified periods. This constraint was a motivation for participants to perform the task as fast as possible. Furthermore, we encouraged participants by additional monetary reward. The three fastest participants could win an additional amount of money.

The experiment consisted of 80 trials, of which 40 trials used the unconstrained condition and 40 trials used the constrained condition. Participants were randomly assigned to ABBA or BAAB order of A and B blocks (with 20 trials per block). The 80 unique block-models, one per trial, were the same for all participants, but their order was randomized, and they could occur in an unconstrained or a constrained conditions.

### Excluded Data

We recorded 1600 trials in the study (20 participants × 80 trials) and excluded from further data analysis 13% of these trials (206 out of 1600): 89 trials (11%) in the unconstrained condition and 117 trials (15%) in the constrained condition. We excluded these trials based on two factors. In 198 trials the subjects did not finish the task in time. We excluded five additional trials as participants made fewer than 3 or more than 24 saccades into the Model Area from outside of the Model Area and thus extremely deviating from other trials.

The model did not appear on the monitor in the constrained condition when a participant made a saccade into the Model Area and then a saccade out of the Model Area before the 0.7 s delay period was over. We detected 255 such instances in the study (∼0.16 per trial). We removed both, the saccade into the Model Area and the return saccade out of the Model Area, as they did not provide any information gain (the Lego-model stayed covered). These removed saccades had no influence on the conclusions of the study.

### Equipment

The experiment was set up as a gaze-contingent paradigm with eye and mouse tracking. We used a desktop mount infrared eye-tracking device (Eyelink 1000, SR Research Ltd., Mississauga, Canada) in remote mode with 500 Hz sampling-rate to track eye-movements. Its standard settings were used to classify fixations, saccades, and blinks. An infrared illuminator module with 890 nm wavelength illuminated the faces of the participants. By applying an adhesive target on the forehead of the participants, head movements were compensated, allowing tracking without head fixation. A calibration procedure with 13 points assured that the average fixation-deviation was below 0.5° visual angle. We showed stimuli on a 60 Hz, 23″ Dell monitor with a resolution of 1920 × 1080 pixels in full-screen mode in a darkened room. Participants had to use a mouse to navigate, rotate and position the blocks and a keyboard was used to navigate the instructions.

## Results

**FIGURE 3:**
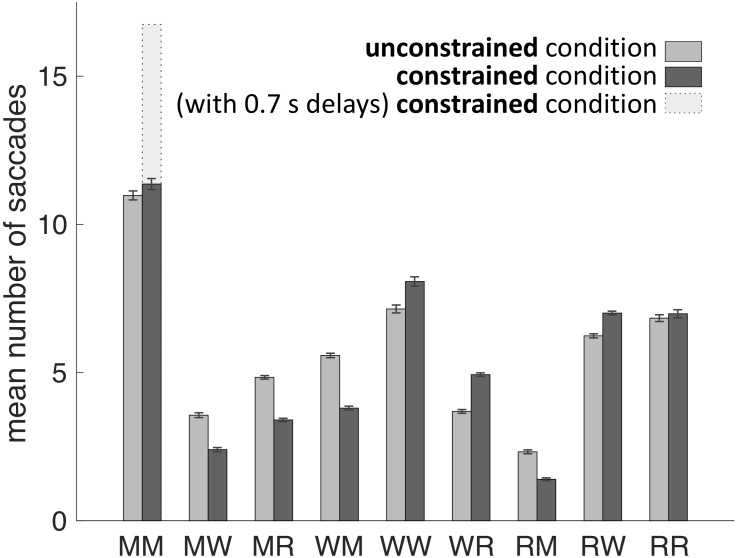
Mean number of saccades per trial between and within the Model Area (M), the Work Area (W), and the Resource Area (R) in unconstrained and constrained conditions for all subjects and all trials. Error bars represent the standard error of the mean. See exact values in Table 1.

**FIGURE 4:**
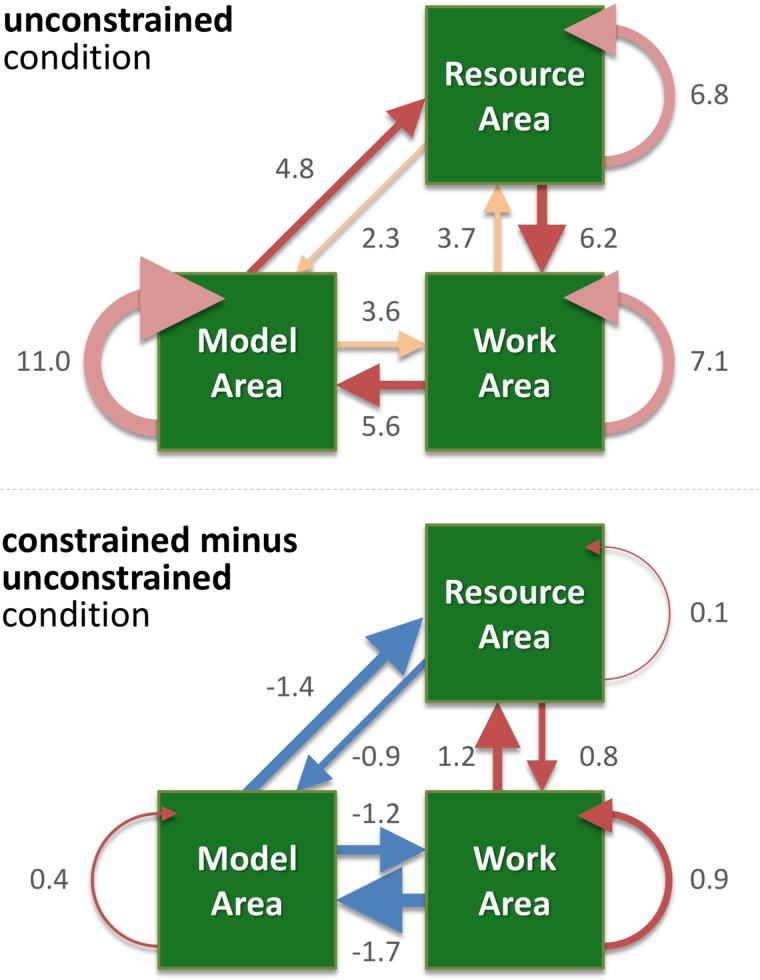
The weighted directed graph shows the mean number of saccades per trial in the unconstrained condition (upper panel) and the difference between the unconstrained and constrained conditions (lower panel). Values represent the mean number of saccades per trial. The graph was built according to the data shown in Figure 3 and Table 1, which also lists the standard errors. Here, in both panels, the width of an arrow represents relative value, i.e., number of occurrences of the respective type of saccade. In the upper panel “unconstrained condition,” different shades of red highlight the RM-MW-WR, MR-RW-WM saccadic circles, and inside the same area saccades (RR, MM, WW). In the lower panel “constrained condition minus unconstrained condition,” blue arrows represent negative values, red – positive values.

**FIGURE 5:**
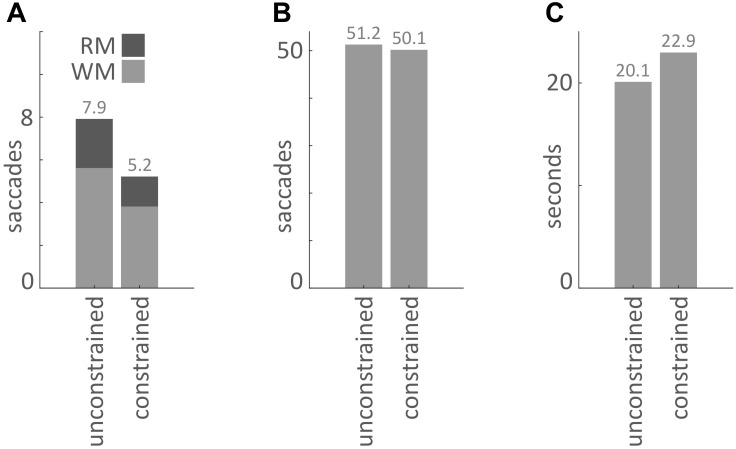
**(A)** Mean number of saccades per trial into the Model Area from the Work Area (WM) and from the Resource Area (RM) for the unconstrained and constrained conditions. **(B)** Mean number of saccades per trial in the unconstrained and constrained conditions. The bar for the constrained condition does not include MM saccades during 0.7 s delays (Figures 2, 3). **(C)** Mean duration of a trial in the unconstrained and constrained conditions. The bar for the constrained condition does not include the 0.7 s delay periods.

Our main hypothesis was that participants would avoid penalized saccades into the Model Area and, therefore, shift the balance between memorization and active gaze toward memorizing more blocks of the models. To answer this question, saccades were classified according to their start- and end-locations (Model Area, Work Area, or Resource Area, abbreviated M, W, and R respectively). We calculated the number of occurrences for all of the nine possible combinations of start and end locations (Figures 3, 4). Figure 5A sums up the main experimental result, i.e., difference between unconstrained and constrained conditions for WM and RM types of saccade. In the unconstrained condition, participants made on average 7.9 saccades into the Model Area (RM and WM) to successfully copy a model of 8 blocks. In the constrained condition participants made on average 5.2 saccades into the Model Area (RM and WM) to successfully copy a model of 8 blocks (Figure 5A). Participants made per trial on average 1.1 saccades less (2%) in the constrained condition (Figure 5B). The mean number of saccades of all types per trial in the unconstrained condition was 51.2 and in the constrained condition 50.1. Please note, the mean number of saccades in the constrained condition does not contain MM saccades during the 0.7 s delay periods. The overall rearrangement of saccades between areas in a trial is shown in Figure 4.

Saccades into the Model Area (RM and WM) would indicate a need to refresh the memory and check the model. Saccades into the Resource Area (MR and WR) would indicate the need to pick up a Lego block. Saccades into the Work Area (MW and RW) would indicate the readiness to place a picked up Lego block, or the need to refresh information about the missing parts of the already constructed Lego model. MM, WW, and RR saccade would be related to inspection of the Lego model or to a choice of a how to progress with building the model.

### Model–Model Saccades

**FIGURE 6:**
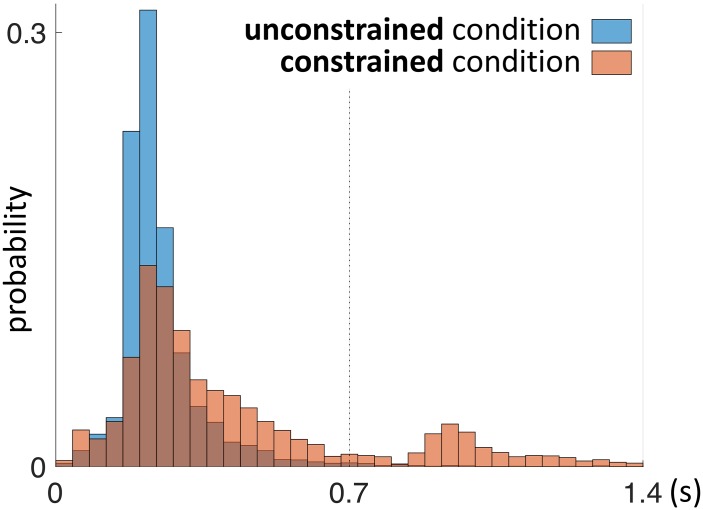
Probability distribution of the duration of the first fixation plus first saccade during the penalty period. This time interval is defined by the end of “Saccade 1” and the end “Saccade 2” as shown schematically in Figure 2. The unconstrained condition is shown in blue, the constrained condition in red. Red bars in the time interval 0–0.7 s represent saccades (“Saccade 2” in Figure 2) which did not gain information about the model, as the model was not yet uncovered during that time interval.

First we address the first saccades within the Model Area (MM, e.g., Saccade 2 in Figure 2) in the constrained condition, as they are directly affected by the delay period until the model is uncovered. With respect to the first MM saccade, even in the absence of relevant information during the 0.7 s delay period of the constrained condition, participants retained an eye-movement dynamic similar to the unconstrained condition (Figure 6). Additionally, we observed a shift in the distribution of fixation durations for the constrained condition toward a second, later peak at 0.92–0.96 s (Figure 6).

The early saccades in the constrained condition did not provide relevant information for the task, because a model appeared only after the delay period. The mean number of MM saccades including MM saccades during the delay periods is equal to 16.8 ± 0.23 (Table 1; Figure 3, MM light bar) and excluding MM saccades during the delay periods is equal to 11.4 ± 0.19 (Table 1; Figure 3, MM dark bar). Discounting the saccades during the delay periods, we observe only minimal changes in the frequency of MM saccades (11.0 ± 0.15 in the unconstrained condition and 11.4 ± 0.19 in the constrained condition). For further comparisons with the unconstrained condition we excluded all saccades within the Model Area (MM) during the delay period of the constrained condition. Summarizing, although visiting the Model Area in the constrained condition incurs a penalty in the form of a delay, we have no indication that it is subsequently sampled with a larger number of MM saccades than in the unconstrained condition.

**Table 1:** Mean number of saccades per trial between and within the Model Area (M), the Work Area (W), and the Resource Area (R) in unconstrained and constrained conditions for all subjects and all trials.

Saccade Type	MM	MW	MR	WM	WW	WR	RM	RW	RR
Unconstrained Condition	11.0 ± 0.15	3.6 ± 0.08	4.8 ± 0.06	5.6 ± 0.08	7.1 ± 0.13	3.7 ± 0.06	2.3 ± 0.07	6.2 ± 0.07	6.8 ± 0.11
Constrained Condition	11.4 ± 0.19	2.4 ± 0.07	3.4 ± 0.05	3.8 ± 0.06	8.1 ± 0.16	4.9 ± 0.06	1.4 ± 0.04	7.0 ± 0.06	7.0 ± 0.14
Constrained condition with MM saccades within 0.7 s delay periods (empty Model Area)	16.8 ± 0.23	–	–	–	–	–	–	–	–


*Errors represent the standard error of the mean.*

### Dependence of Distribution of Saccades on the Condition

We proceed with a statistical analysis of the overall distribution of saccades in the two conditions. Saccades were tested with the saccades during the 0.7 s delay periods removed. The two-way analysis of variance (ANOVA) of factors Condition and Type of Saccades found statistical significance for the factor Type of Saccades (SS = 2508, SSTotal = 3454 *df* = 8, *F* = 127.21, *p* < 0.001). It shows that not all types of saccades are equally probable. The factor Condition was not statistically significant (SS = 2.5, SSTotal = 3454, *df* = 1, *F* = 1.01, *p* = 0.3). This shows that subjects on average did not make significantly more or fewer saccades between the unconstrained and constrained conditions. Two-sample *t*-test (unconstrained and constrained conditions) did not reject the null hypothesis at the 5% significance level that the mean numbers of saccades per trial are identical in unconstrained and constrained conditions (Figure 5B). Thus, we do not have an indication that the experimental manipulation changed the total number of saccades, although it does not prove the equivalence of the total number of saccades either. Importantly, the interaction of factors Condition and Type of Saccades was statistically significant (SS = 101, SSTotal = 3454 *df* = 8, *F* = 5.13, *p* < 0.001). This means that the probability of making a certain type of saccade depends on the condition. We tested null hypothesis for the number of saccades in the unconstrained and constrained condition. For all types of saccades (MW/MR/WM/WW/WR/RM/RW) with the exception of MM and RR saccades we observe a highly significant difference (Table 2). Two-sample *t*-test (unconstrained and constrained conditions) did not reject the null hypothesis at the 5% significance level for MM, and RR types of saccades but rejected the null hypothesis for the other types of saccades. This indicates that the probability of occurrence of all types of saccades from an area to another has changed.

**Table 2:** Two-sample *t*-test for the number of saccades in the unconstrained and constrained conditions (we excluded MM saccades in the penalty intervals).

	MM	MW	MR	WM	WW	WR	RM	RW	RR
p	0.11	<0.01	<0.01	<0.01	<0.01	<0.01	<0.01	<0.01	0.41


*Data from 711 valid trials in the unconstrained condition and 683 valid trials in the constrained condition.*

We visualize the complex changes of viewing behavior during the task by a weighted directed graph. It shows the mean number of saccades per trail within and between the areas for the unconstrained condition (Figure 4, upper panel). In Figure 4 (lower panel) the difference between the unconstrained and constrained conditions reveals the effect of the delay period of sampling of visual information from the Model Area. Specifically, participants reduced sampling of the Model Area in the constrained condition (blue arrows in Figure 4), and instead, performed more saccades within each area and between the Work Area and the Resource Area (red arrows).

**FIGURE 7:**
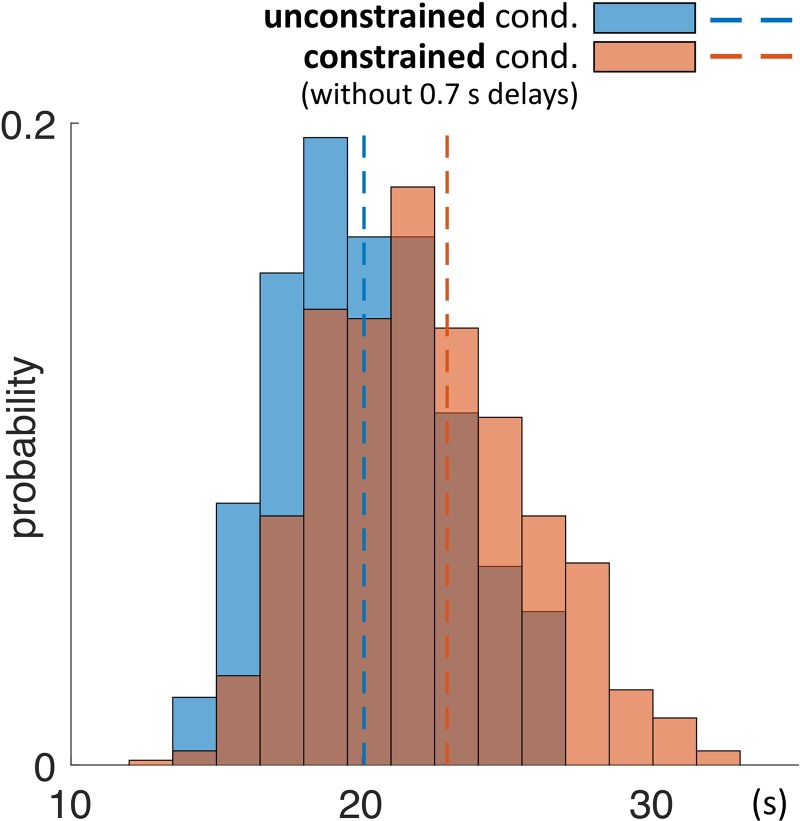
Probability distribution of duration of trials in the unconstrained and constrained conditions. Trials ended automatically when participants correctly finished copying the entire model. Duration of trials in the constrained condition (red) does not include the 0.7 s penalty-delay periods. Bin width is equal to 1.5 s.

As a next step we investigated the mean duration of trials in each condition. The mean duration of a trial in the unconstrained condition was 20.1 s ± 0.1 s and in constrained condition 22.9 s ± 0.1 s (Table 3 and Figures 5C, 7). Importantly, these numbers do not include the 0.7 s delay periods of the constrained condition. A Kolmogorov–Smirnov test rejected the null hypothesis that duration of trials in the unconstrained and constrained conditions originate from the same continuous distribution (*p* < 0.001) (Figure 7). Thus, in the constrained condition we observed significantly longer trial durations – 2.9 s (14%) (Table 3 and Figures 5C, 7), even when discounting for the penalty-delay periods.

**Table 3:** Mean duration of trials, mean duration of dwelling time in areas, and mean total duration of saccades during a trial.

	Mean duration of a trial	Dwelling time in Model Area	Dwelling time in Work Area	Dwelling time in Resource Area	Time taken by all Saccades
Unconstrained condition	20.07 ± 0.11 s	4.88 ± 0.05 s	8.28 ± 0.07 s	4.48 ± 0.04 s	2.43 ± 0.03 s
Constrained condition^∗^	22.93 ± 0.13 s	5.90 ± 0.06 s	9.78 ± 0.08 s	4.76 ± 0.05 s	2.49 ± 0.03 s


*Error terms represent the standard error of the mean. ^∗^Constrained condition without the 0.7 s delay periods.*

The dwelling time, i.e., the total time fixations were directed to a specific area, selectively increased on the Model Area, that generally supports the idea of shifting to fewer samples but longer/more meaningful samples. The durations also increase on the Work/Resource Areas, that is consistent with a general increase in cognitive load. Please note that the total time taken by saccades is similar in both conditions (Table 3).

## Discussion

The presence of a task leads to a strong influence on eye-movement-behavior where the task mainly determines where participants look (Rothkopf et al., 2007; Betz et al., 2010). In this study, we have demonstrated that not only a task but also a price for a certain type of saccades significantly influenced eye-movement-behavior of participants. We investigated whether a change of the price of a certain type of saccades shifts the balance between performing saccades and storing information in memory. We observed a reduced regularity of visiting the area in sequence of Model – Resource – Work Area. Instead, the number of visits in the Model Area was reduced and the Resource and Work Areas were more often visited in alternation. However, the fewer visits to the Model Area were not more thorough, but comprised about the same number of useful fixations while the model was visible as in the unconstrained condition. As a consequence, the total time needed for completion of the block copying task increased, even when discounting for the penalty delay periods at the beginning of a visit to the Model Area. This means, that just enduring the penalty delay period with unchanged visual behavior would have lead to higher performance. Thus, participants’ behavior significantly changed toward increased use of working memory when we introduced the price of saccades, but it was not adaptive in the sense of optimal performance.

Humans seek to minimize the use of short-term memory by serializing tasks with eye movements, thus most of visual information is acquired just prior to its use (Ballard et al., 1995). Eye movements serialize complex operations by deploying foveal acuity to objects 500 ms before reaching (Pelz and Canosa, 2001), as required by the task (Rothkopf et al., 2007).

It is unlikely that a complete representation of the visual scene is maintained in visual working memory (Marr and Vaina, 1982; Nakayama, 1990). Further studies showed a limitation of visual working memory in the form of a limited capacity of 3–7 items (Luck and Vogel, 1997; Cowan, 2001; Todd and Marois, 2004). In our experiment, a model consisted of 8 blocks. Each block was characterized by several features (color, size, orientation, and location), making it hard for participants to remember more than a few blocks at once. Indeed, our results suggest that participants made a saccade to obtain features of each single block in the unconstrained condition (7.9 saccades into the Model Area per trial – WM and RM). The change of the strategy in the constrained condition (5.2 saccades into the Model Area per trial) showed that subjects adapted to the changing environment. They sampled the Model Area less often. However, the sampling time and the number of saccades within in the Model Area after expiration of the delay period was not increased. This suggests that sampling during visits of the Model Area was unchanged. In summary, participants were able to dynamically react to the trade-off between sampling of visual information and using of working memory, but not optimally.

This study focuses on the key comparison, either the model is revealed instantly or with a delay. All other aspects are identical. Introducing an additional condition, e.g., the model is always visible would introduce additional complexity. Participants might try to utilize peripheral vision and reduce the number of checks in the Model Area. They might try to fixate close to but not within the Model Area. This is an interesting aspect, but additional to the question addressed here.

We have chosen a mid-level complexity of the model (eight blocks). Simpler models could tempt subjects to memorize all in one go, more complex models could lead to an increasing fraction of control fixations. Although such effects are interesting, they do not directly relate to the topic of the present study. Moreover, we could introduce a parameter of distraction, e.g., auditory or visual distractors (Schut et al., 2017), or a parallel subtask. That would allow for building a more precise and comprehensive model of the balance between memorization and active sampling of visual information. Then, it could be possible to see a point of a strategy shift and dynamic adoption of eye-movement behavior.

Next, we consider differences between the two conditions with respect to the relative timing of fixation onset and onset of the visual information in the Model Area. In the unconstrained condition a saccadic eye movement toward the Model Area had to be detected and the display of a Lego-model be switched on. The 60 Hz refresh rate of the monitor may result in a small delay of screen refresh (∼17 ms). We use such a gaze contingent setup routinely and tested it thoroughly (Ehinger et al., 2018). Further, in the present study the region of interest, i.e., the Model Area, was large and distant from the other areas, which makes detection of relevant fixations particularly fast and easy. Given the spatial setup of the Resource, Work, and Model Areas and that most saccades target the center of the Model Area we estimate that only a small fraction of fixations in the Model Area is started before the model is displayed (refresh of the monitor). A study on eye-movement control during scene-viewing (Henderson and Graham, 2008; Henderson and Smith, 2009) demonstrated that if in a gaze contingent paradigm a scene is only revealed a short delay after fixation onset, then a part of such fixations also last approximately that delay longer. Overall, a few fixations after late detected saccades might last a few milliseconds longer. Compared to the total data set, the length of the trials and the effect size, such a small effect seems negligible. In the constrained condition, the model appeared during an ongoing fixation. The delay of 700 ms itself was excluded from the analysis. Theeuwes et al. (1998) showed that for delays of at least 600 ms, capture effects by the appearance of a task-irrelevant onset can be overcome when observers have sufficient time to attend and program an eye movement to the location of a subsequent target stimulus. Another study (Walshe and Nuthmann, 2014) demonstrated “no immediate decrease of fixation durations when luminance is increased.” In our paradigm, the visible model is not a distractor, but the task relevant scene; the model is shown at a high contrast level, which allows subjects to carry out the task of memorizing the model. Therefore, the fact that model appeared during an ongoing fixation should not contribute to the mean duration of the trials in the constrained condition (Figure 5C). Overall, the relative timing of stimulus onset and fixation onset can not explain the results presented here.

A different study (Gray et al., 2005) used a similar block-copying task to the one in the present study, in order to build a computational model of trading off interaction-intensive vs. memory-intensive strategies in the Adaptive Control of Thought-Rational (ACT-R) cognitive architecture (Anderson and Lebiere, 2014). The authors reported that the ACT-R framework (at the time of the study) was not capable to explain human adaptation to the task environment. Behavioral results in that study demonstrated that human subjects were trading off interaction-intensive for more memory-intensive strategies. However, the behavioral part in their study was conducted and analyzed without eye-tracking data.

Theories of embodied cognition postulate the world as an external memory (Clark and Chalmers, 1998). Under this view, instead of storing visual information in working memory the information may be equally retrieved by appropriate eye movements (Van Gompel, 2007). We designed this study to quantify the trade-off between the use of working-memory and eye movements during purposeful actions. The cost for a new sample of visual information that participants had to “pay” was a short delay until a model of blocks appeared on a screen. Participants’ behavior significantly changed toward increased use of working memory when we introduced the price for a certain type of saccades. Thus, our results supported the view that postulated the world as an external memory.

## Author Contributions

FS, CR, and PK study conception and design. FS acquisition of data. AM and FS analysis and interpretation of data, drafting of manuscript. AM, PK, and CR critical revision.

## Conflict of Interest Statement

The authors declare that the research was conducted in the absence of any commercial or financial relationships that could be construed as a potential conflict of interest.
